# Severe Mitral Regurgitation Requiring Transjugular Transcatheter Edge-to-Edge Repair With the PASCAL System

**DOI:** 10.1016/j.jaccas.2024.102573

**Published:** 2024-10-02

**Authors:** Peter T. Hu, Brian Whisenant

**Affiliations:** Intermountain Medical Center, Salt Lake City, Utah, USA

**Keywords:** mitral transcatheter edge-to-edge repair, PASCAL, primary mitral regurgitation, severe mitral regurgitation, transjugular approach

## Abstract

In severely symptomatic patients with primary severe mitral regurgitation at high or prohibitive surgical risk, mitral transcatheter edge-to-edge repair is a reasonable strategy. We present a successful case of right internal jugular vein access using PASCAL mitral valve repair (Edwards Lifesciences) in a patient with prohibitive risk for surgical mitral repair.

## Graphical Abstract

**Figure:**
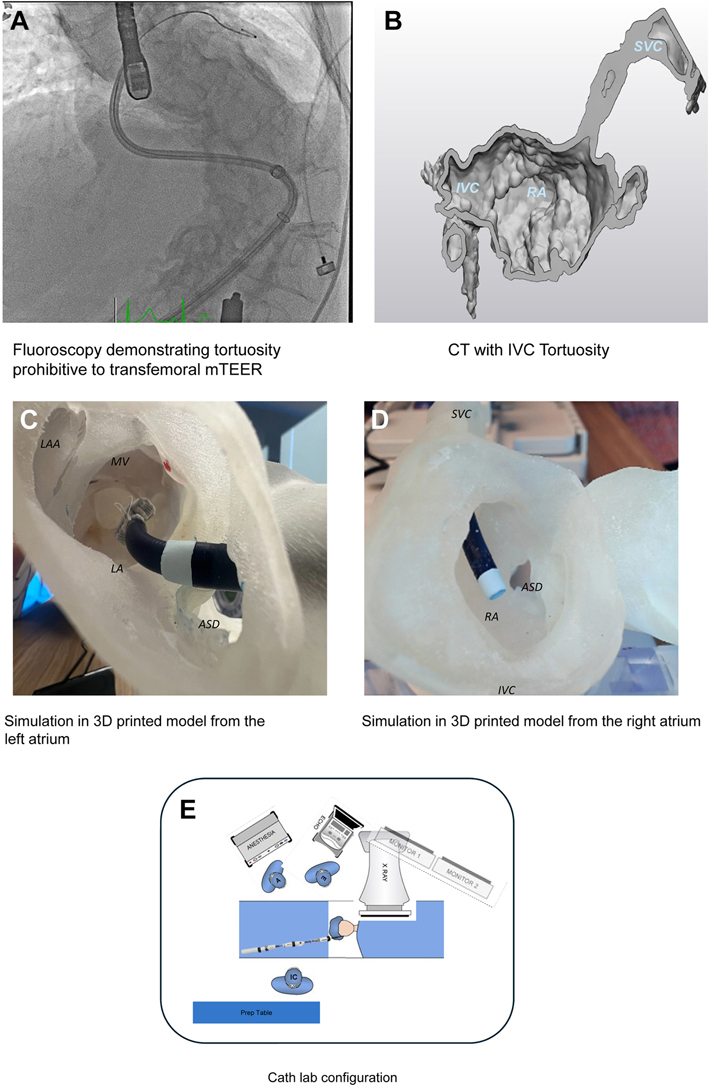

Primary mitral valve regurgitation (MR) is a mechanical problem of leaflet coaptation. When surgical risk for mitral valve repair is deemed prohibitive, patients with severe, symptomatic primary MR derive benefit from mitral transcatheter edge-to-edge repair (mTEER).^1^^,^^2^

**Take-Home Messages**

• Transjugular venous access is a feasible alternative for mitral transcatheter edge-to-edge repair in patients with complex anatomy and severe mitral regurgitation.
• This case highlights the importance of preprocedural planning, technical expertise, and a dedicated heart team in cases where traditional transfemoral veinous access for mitral transcatheter edge-to-edge repair may not be possible.

A 71-year-old woman with a history of McArdle glycogen storage disease, severe scoliosis, and chronic obstructive pulmonary disease on oxygen presented with exercise intolerance. A transesophageal echocardiogram (TEE) demonstrated a flail P2 segment with severe MR and normal biventricular function (Video 1A). Coronary angiography did not show obstructive coronary artery disease. The patient was evaluated by cardiac surgery for mitral valve repair, but the risk was deemed prohibitive, and she was referred for mTEER.

MitraClip G4 (Abbott) was attempted via the right femoral vein. With severe scoliosis and inferior vena cava (IVC) tortuosity (Figure 1A), transeptal access was difficult. Advancement of the MitraClip steerable guide sheath collapsed the interatrial septum, resulting in a large atrial septal defect (ASD). While advancing the MitraClip G4 system to the left atrium, the patient developed cardiac tamponade, requiring pericardiocentesis, and the procedure was aborted. The patient recovered with NYHA functional class III symptoms, and the heart team pursued right internal jugular vein (IJ) mTEER with the PASCAL system (Edwards Lifesciences).

**Figure 1:**
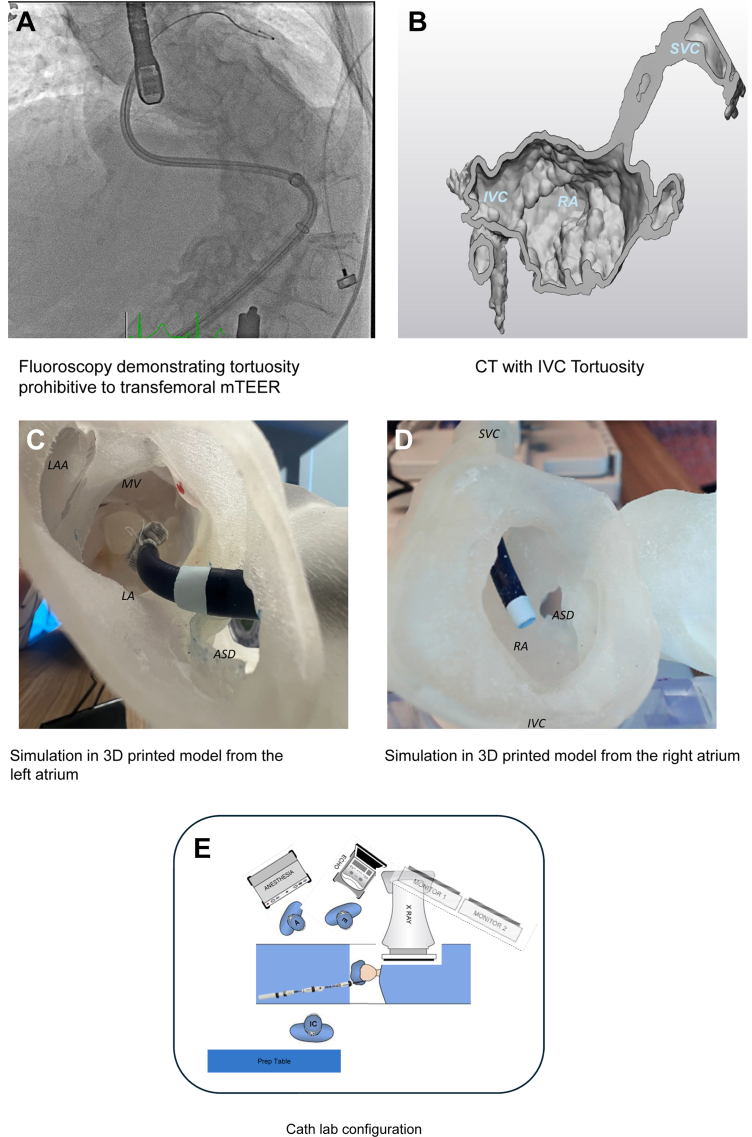
Preprocedural Planning (A) Fluoroscopy demonstrating severe scoliosis and tortuosity of the inferior vena cava from a right common femoral vein approach. (B) Computed tomography–generated 3-dimensional image demonstrating the IVC tortuosity as well as the SVC trajectory. (C) A 3-dimensional printed model demonstrating right transjugular access with the delivery sheath and device from the left atrium. (D) A 3-dimensional printed model demonstrating right transjugular access with the delivery sheath and device from the right atrium. (E) Catheter laboratory configuration to perform internal jugular vein mitral transcatheter edge-to-edge repair. A = anesthesiologist; ASD = atrial septal defect; E = echocardiologist; ECHO = echocardiography; IC = interventional cardiologist; IVC = inferior vena cava; LA = left atrium; LAA = left atrial appendage; MV = mitral valve; RA = right atrium; SVC = superior vena cava.

The PASCAL device may be uniquely suited to IJ access. The PASCAL delivery catheter telescopes through the PASCAL guide sheath in a nonkeyed fashion. Independent flexion and rotation of the 2 steerable catheters yield infinite combinations. The device can be closed and flexed as it exits the sheath. The implant can be elongated, enabling easy retrieval from the left ventricle with reduced risk of chordal entanglement.

Cardiac computed tomography (Figure 1B) was used to create a 3-dimensional (3D) printed model of the patient’s heart. IJ delivery of a PASCAL Ace device was simulated using the 3D model (Figures 1C and 1D). Various configurations were explored until an S-curve approach provided an ideal trajectory and height above the valve.

Three months following the aborted MitraClip procedure, the patient presented for redo mTEER with the PASCAL Precision system. TEE confirmed the P2 flail/severe MR. The delivery sheath was advanced to the IVC, withdrawn, and flexed under TEE guidance, passing through the iatrogenic ASD. After advancing the PASCAL implant to the left atrium, the steerable catheter was flexed toward the mitral valve under fluoroscopic and TEE guidance with an S-like trajectory, opposite of the guide catheter (Video 1B). Unlike a transfemoral procedure, advancing and retracting the system does not translate to a medial/lateral position. Flexion determined the medial/lateral position. The PASCAL Ace implant was positioned above the valve in an S-shaped fashion (Video 1B). When compared with transfemoral TEER, clockwise and counterclockwise rotation of the steerable catheter resulted in roughly opposite anatomic steering, that is, clockwise rotation moved the implant anteriorly. Video 1C demonstrates adequate grasp of the A2/P2 segments of the mitral valve utilizing 3D multiplane reconstruction. There was mild residual MR on 2-dimensional TEE with color Doppler biplane (Video 1D). After successful implantation of the PASCAL Ace implant, the ASD was closed with a 20-mm Amplatzer Septal Occluder (Abbott). No complications were noted. The patient noted significant improvement.

In conclusion, we present a patient with severe primary MR who required an IJ approach for mTEER using the PASCAL Precision system.

## Funding Support and Author Disclosures

Dr Whisenant has served as a consultant for Edwards Lifesciences and Abbott Vascular. Dr Hu has reported that he has no relationships relevant to the contents of this paper to disclose.

## Appendix

Video 1A Periprocedural Imaging (A) A 0° mid-esophageal transesophageal echocardiogram demonstrating posterior leaflet flail with severe, eccentric anteriorly directed MR. (B) Fluoroscopy showing the PASCAL Ace implant positioned above the valve in an S-shaped fashion, replicating the approach from the printed model. (C) Intraprocedural 3D multiplanar reconstruction of the PASCAL Ace implant demonstrating capture of the mitral leaflets within the paddles and clasps. (D) A 2-dimensional image with color Doppler and biplane from the commissural view on the left and long-axis view on the right demonstrating mild residual MR.

Video 1B XXXX (A) A 0° mid-esophageal transesophageal echocardiogram demonstrating posterior leaflet flail with severe, eccentric anteriorly directed MR. (B) Fluoroscopy showing the PASCAL Ace implant positioned above the valve in an S-shaped fashion, replicating the approach from the printed model. (C) Intraprocedural 3D multiplanar reconstruction of the PASCAL Ace implant demonstrating capture of the mitral leaflets within the paddles and clasps. (D) A 2-dimensional image with color Doppler and biplane from the commissural view on the left and long-axis view on the right demonstrating mild residual MR.

Video 1C XXXX (A) A 0° mid-esophageal transesophageal echocardiogram demonstrating posterior leaflet flail with severe, eccentric anteriorly directed MR. (B) Fluoroscopy showing the PASCAL Ace implant positioned above the valve in an S-shaped fashion, replicating the approach from the printed model. (C) Intraprocedural 3D multiplanar reconstruction of the PASCAL Ace implant demonstrating capture of the mitral leaflets within the paddles and clasps. (D) A 2-dimensional image with color Doppler and biplane from the commissural view on the left and long-axis view on the right demonstrating mild residual MR.

Video 1D XXXX (A) A 0° mid-esophageal transesophageal echocardiogram demonstrating posterior leaflet flail with severe, eccentric anteriorly directed MR. (B) Fluoroscopy showing the PASCAL Ace implant positioned above the valve in an S-shaped fashion, replicating the approach from the printed model. (C) Intraprocedural 3D multiplanar reconstruction of the PASCAL Ace implant demonstrating capture of the mitral leaflets within the paddles and clasps. (D) A 2-dimensional image with color Doppler and biplane from the commissural view on the left and long-axis view on the right demonstrating mild residual MR.
